# Molecular Investigation of Chicken Acid-Sensing Ion Channel 1 β11-12 Linker Isomerization and Channel Kinetics

**DOI:** 10.3389/fncel.2021.761813

**Published:** 2021-11-24

**Authors:** Matthew L. Rook, Anna Ananchenko, Maria Musgaard, David M. MacLean

**Affiliations:** ^1^Graduate Program in Cellular and Molecular Pharmacology and Physiology, University of Rochester Medical Center, Rochester, NY, United States; ^2^Department of Chemistry and Biomolecular Sciences, University of Ottawa, Ottawa, ON, Canada; ^3^Department of Pharmacology and Physiology, University of Rochester Medical Center, Rochester, NY, United States

**Keywords:** gating, desensitization, acid-sensing ion channels, ASICs, molecular dynamics

## Abstract

Structures of the trimeric acid-sensing ion channel have been solved in the resting, toxin-bound open and desensitized states. Within the extracellular domain, there is little difference between the toxin-bound open state and the desensitized state. The main exception is that a loop connecting the 11th and 12th β-strand, just two amino acid residues long, undergoes a significant and functionally critical re-orientation or flipping between the open and desensitized conformations. Here we investigate how specific interactions within the surrounding area influence linker stability in the “flipped” desensitized state using all-atom molecular dynamics simulations. An inherent challenge is bringing the relatively slow channel desensitization and recovery processes (in the milliseconds to seconds) within the time window of all-atom simulations (hundreds of nanoseconds). To accelerate channel behavior, we first identified the channel mutations at either the Leu414 or Asn415 position with the fastest recovery kinetics followed by molecular dynamics simulations of these mutants in a deprotonated state, accelerating recovery. By mutating one residue in the loop and examining the evolution of interactions in the neighbor, we identified a novel electrostatic interaction and validated prior important interactions. Subsequent functional analysis corroborates these findings, shedding light on the molecular factors controlling proton-mediated transitions between functional states of the channel. Together, these data suggest that the flipped loop in the desensitized state is stabilized by interactions from surrounding regions keeping both L414 and N415 in place. Interestingly, very few mutations in the loop allow for equivalent channel kinetics and desensitized state stability. The high degree of sequence conservation in this region therefore indicates that the stability of the ASIC desensitized state is under strong selective pressure and underlines the physiological importance of desensitization.

## Introduction

The majority of ligand-gated ion channels (LGICs) undergo a process of desensitization wherein channels enter a long-lived non-conducting state in the continued presence of agonist (Katz and Thesleff, 1957). Thus, desensitization shapes the time course of ion flux and may also play a protective role when ligand clearance is compromised (Jones and Westbrook, 1996; Christie et al., 2010). Desensitized channels must ultimately recover from this non-conducting state before they can be activated again, and, like desensitization, the recovery time course also influences the physiological roles of LGICs (Chen et al., 2018). The era of structural biology has provided many insights and hypotheses regarding the molecular basis of desensitization for numerous LGICs (Plested, 2016; Noviello et al., 2021; Yu et al., 2021) including acid-sensing ion channels (ASICs) (Gonzales et al., 2009; Baconguis and Gouaux, 2012; Baconguis et al., 2014; Yoder et al., 2018; Yoder and Gouaux, 2020).

**FIGURE 1:**
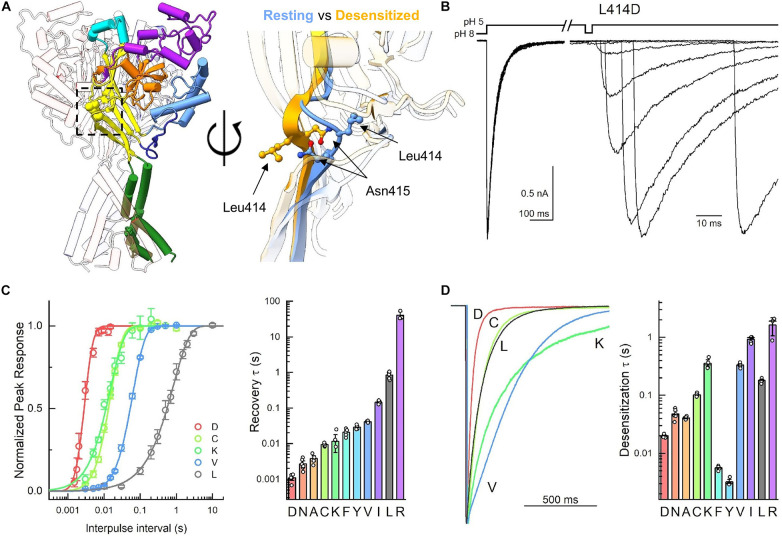
Mutations to Leu414 have substantial impact on channel kinetics. (**A**, left) Trimeric structure of the resting state with individual domains colored (PDB: 6VTL). (**A**, right) Zoom in of the β11-12 linker in overlay of resting (6VTL, blue) and desensitized states (6VTK, orange). **(B)** Example outside-out patch recordings of L414D during recovery from desensitization protocol. Note the change in time scale in the example traces. **(C)** Summary recovery time course (left) and time constants (right) for Leu414 mutant panel from this and previous work. **(D)** Example traces (left) and summary of time constants (right) for desensitization decays of Leu414 mutant panel. Symbols represent individual patches and error bars are SEM. Differences in both desensitization and recovery kinetics for all mutations were statistically significant from wild type. Some data reproduced from Rook et al. (2020) under Creative Commons Attribution License (https://creativecommons.org/licenses/by/4.0/).

Acid-sensing ion channels are trimeric sodium-selective channels found throughout the brain and body (Boscardin et al., 2016). In response to a rapid drop in extracellular pH, ASICs activate and subsequently desensitize nearly completely with time constants ranging from 100 ms to 2 s, depending on the species, subunit and pH stimulus (Grunder and Pusch, 2015; Rook et al., 2021b). Recent structures of the resting, toxin-stabilized open and desensitized states of chicken ASIC1 (cASIC1) showed that the main difference in the ASIC extracellular domains of desensitized channels, relative to resting and open channels, is the isomerization of two amino acid residues linking the 11th and 12th β-strands (Yoder et al., 2018; Rook et al., 2021b). In the resting and toxin-stabilized open state, the first amino acid in this linker (Leu414) points up and away from the middle of the channel while the second amino acid (Asn415) points downward (Figures 1A,B). In the desensitized structures, this linker has isomerized or flipped such that Leu414 now points inward and Asn415 outward (Figure 1B). This was hypothesized to reflect a “molecular clutch” (Yoder et al., 2018). When the clutch is engaged, conformational changes in the extracellular domain can propagate to the pore and drive activation. However, once the clutch disengages (i.e., the linker flips), conformational changes are no longer effectively coupled to the pore. This enables the extracellular domain to remain in an “active” state while the pore can close, preventing continued ion influx under acidic conditions (Yoder et al., 2018). This elegant mechanism explains earlier functional data and was recently supported by additional mutations, simulations and state-dependent crosslinking (Rook et al., 2020). The goal of the present work was to further probe the molecular determinants of linker flipping using molecular dynamics simulations in conjunction with fast-perfusion electrophysiology.

To do this, we measured the kinetics of desensitization and recovery for a panel of mutations at both the Leu414 and Asn415 positions. We reasoned that the fastest mutations would have an increased propensity for motion of the β11-12 linker, thus potentially allowing us to observe linker dynamics within the timeframe of MD. Thus, the fastest mutants were used for subsequent all-atom molecular dynamics simulations to study the behavior of the β11-12 linker. Our simulations identified previously characterized electrostatic interactions as well as a novel hydrogen bond interaction that controls the stability of the ASIC desensitized state. Taken together, these data deepen our understanding of the molecular determinants of ASIC desensitization while advancing new metrics for the analysis of these transitions.

## Materials and Methods

### Cell Culture, Mutagenesis and Transfection

Human Embryonic Kidney 293 ASIC1 knockout (KO) cells (Rook et al., 2021a) were maintained in Dulbecco’s Modification of Eagle’s Medium (DMEM) with 4.5 g/L glucose, L-glutamine and sodium pyruvate (Corning/Mediatech, Inc.) or Minimum Essential Medium (MEM) with Glutamax and Earle’s Salts (Gibco), supplemented with 10% FBS (Atlas Biologicals) and penicillin/streptomycin (Invitrogen). Cells were passaged every 2–3 days when approximately 90% confluence was achieved. Cells were plated on tissue culture treated 35 mm dishes, transfected 24–48 h later and recorded from 24 to 48 h post-transfection. Cells were transiently transfected with wild type or mutations of full length cASIC1 (in pcDNA3.1(+)) along with eGFP using an ASIC:eGFP ratio of 2.5:1 μg of cDNA per 10 mL of media. Transfections were performed using polyethylenimine 25k (PEI 25k, Polysciences, Inc.) following manufacturer’s instructions, with media change at 6–8 h. Mutations were introduced using site-directed mutagenesis PCR and confirmed by sequencing (Fisher Scientific/Eurofins Genomics).

### Electrophysiology and Fast Perfusion

Culture dishes were visualized using a 20× objective mounted on a Nikon Ti2 microscope with phase contrast. A 470 nm LED (Thorlabs) and dichroic filter cube were used to excite GFP and detect transfected HEK cells. Outside-out patches were excised using heat-polished, thick-walled borosilicate glass pipettes of 3–8 MΩ resistance. The pipette internal solution contained (in mM) 135 CsF, 33 CsOH, 11 EGTA, 10 HEPES, 2 MgCl_2_, and 1 CaCl_2_ (pH 7.4). External solutions with a pH greater than 7 were composed of (in mM) 150 NaCl, 20 HEPES, 1 CaCl_2_, and 1 MgCl_2_ with pH values adjusted to their respective values using NaOH. For solutions with a pH lower than 7, HEPES was replaced with MES at the same concentration (20 mM). All recordings were performed at room temperature with a holding potential of −60 mV using an Axopatch 200B amplifier (Molecular Devices). Data were acquired using AxoGraph software (AxoGraph) at 20 kHz, filtered at 10 kHz and digitized using a USB-6343 DAQ (National Instruments). Series resistance was routinely compensated by 90–95% where the peak amplitude exceeded 100 pA. Rapid perfusion was performed using home-built, triple-barrel application pipettes (Vitrocom), manufactured following an established protocol (MacLean, 2015). Translation of application pipettes was achieved using a piezo translator (P601.40 or P212.80, PI) mounted to a manual manipulator and driven by a voltage power supply (E505.00 or E-471.20, PI). Voltage commands to the piezo were first low-pass filtered (eight-pole Bessel; Frequency Devices) at 50–100 Hz. Solution exchange was routinely measured at the end of each patch recording using open tip currents with exchange times ranging from 250 to 500 μs.

### Molecular Dynamics Simulations

Molecular dynamics simulations were performed using a cASIC1 structure solved at 3 Å resolution in a proposed desensitized state (PDB ID: 4NYK) (Gonzales et al., 2009). In this crystal structure, residues 42–455 were resolved, of which 23 residues had missing atoms, which were modeled using MODELLER 9v20 (Sali and Blundell, 1993). Missing residues at the N- and C-termini were disregarded. Bound chloride ions and crystallographically resolved water molecules were retained. The orientation of the structure for placement in the lipid bilayer was obtained from the Orientations of Proteins in Membranes (OPM) database (Lomize et al., 2012). Disulfide bonds for each chain were maintained between the following cysteine pairs: C94-C195, C173-C180, C291-C366, C309-C362, C313-C360, C322-C344, and C324-C336. All residues were kept in their standard ionization state. To construct the simulation system, the protein was embedded in a 120 × 120 Å POPC bilayer using the CHARMM-GUI membrane builder (Jo et al., 2008), with a box length of 147 Å. The system was solvated with TIP3P water molecules and a NaCl concentration of 150 mM.

The L414D and N415G mutant systems were constructed in the same way, with mutations selected in the CHARMM-GUI interface.

The simulations were performed using GROMACS 2019.4 (Abraham et al., 2015) and the CHARMM36m forcefield was applied (Huang and MacKerell, 2013). All system constructs were minimized for 5000 steps or until a maximum force of 1000 kJ mol^–1^ nm^–1^ on any atom was reached. As per the default CHARMM-GUI method, the systems were then equilibrated in six steps. The first three steps were 125 ps long with a timestep of 1 fs. The final three steps were 500 ps long with a timestep of 2 fs. Following the default CHARMM-GUI equilibration, position restraints were gradually lifted with each equilibration step. The final production run had a timestep of 2 fs and was performed for 5 × 500 ns for each system, with the starting velocities of each repeat randomized prior to equilibration. Periodic boundary conditions were applied and all systems were simulated in the NPT ensemble. The Verlet cut-off scheme was used throughout all steps with a force-switch modifier starting at 10 Å and a cut-off of 12 Å. The particle mesh Ewald (PME) method (Darden et al., 1993; Essmann et al., 1995) was used for long-range electrostatics and a cut-off of 12 Å was used for short-range electrostatics. For the equilibration steps, a temperature of 310 K was maintained using the Berendsen thermostat (Berendsen et al., 1984). The Berendsen barostat (Berendsen et al., 1984) was used to maintain a pressure of one bar for the last four steps of equilibration using semi-isotropic pressure coupling. For the production run, the Nose-Hoover thermostat (Nose, 1984; Hoover, 1985) was used to maintain temperature at 310 K and the Parrinello-Rahman barostat (Parrinello and Rahman, 1981) was used to maintain pressure at one bar using semi-isotropic pressure coupling. Covalent bonds including hydrogen atoms were constrained using the LINCS algorithm (Hess, 2008).

Analysis was performed using MDAnalysis tools (Michaud-Agrawal et al., 2011; Gowers et al., 2016) as well as in-house scripts. Structural figures prepared using VMD (Humphrey et al., 1996) and Chimera (Pettersen et al., 2004).

### Sequence Alignments

Amino acid sequences for ASICs from evolutionarily distant organisms (Lynagh et al., 2018) were obtained directly from Dr. Lynagh. Sequences for mammalian, avian, amphibian and fish were obtained from NCBI. Sequences were aligned using Muscle (Edgar, 2004) with default settings. The percent conservation, logo plot and consensus sequence was calculated using Jalview (Waterhouse et al., 2009) and illustrated using Chimera (Meng et al., 2006).

### Statistics and Data Analysis

Current desensitization decays were fitted using exponential decay functions in Clampfit (Molecular Devices). For recovery from desensitization experiments, the piezo command voltage was split and re-directed as an input signal. The resulting piezo “mirror” signal was used to define conditioning and test pulse epochs. A custom script in Matlab (Mathworks) was used to detect peaks within each epoch and normalize the test pulse peak to the conditioning pulse. OriginLab (OriginLab Corp.) was used to fit the normalized responses to:


(1) $$I_{t}={\left(1-e^{\left(\frac{-t}{\tau }\right)}\right)}^{m}$$

where I_*t*_ is the fraction of the test peak at an interpulse interval of *t* compared to the conditioning peak, τ is the time constant of recovery and *m* is the slope of the recovery curve. Each protocol was performed between 1 and 3 times on a single patch, with the resulting test peak/conditioning peak ratios averaged together. For steady-state desensitization (SSD) curves, peak currents within a patch were normalized to the peak response evoked by pH 5.5 and fit to:


(2) Ix=1(1+10((pH50−pHx)nH))

where *I*_*x*_ is the current obtained using a given conditioning pH value X, *pH*_50_ is the pH yielding half maximal response and *n*_*H*_ is the Hill slope. For recovery experiments and SSD, patches were individually fit and averages for the fits were reported in the text. For all experiments, N was taken to be a single patch and error is reported as SEM. Unless otherwise state, non-parametric two-tailed, unpaired randomization tests with 100,000 iterations were implemented in Python to assess statistical significance. Bonferroni-Holm corrections for multiple comparisons were used and the adjusted *P*-values are reported in either text, figure legend or Table 1. Statistical comparisons of recovery from desensitization and SSD were based on differences in recovery time constant and pH_50_, respectively.

**TABLE 1:** Time constants of desensitization and recovery.

Mutation	τ _*des*_ (ms)	τ _*des, fast*_ (ms) [% area]	τ _*des, slow*_ (ms) [% area]	Adj. *P*-value vs. wt	τ _*rec*_ (ms)	m_*rec*_	Adj. *P*-value vs. wt	*n*
WT	180 ± 6	–	–		840 ± 90	0.96 ± 0.05	–	5
A83G	31 ± 2	–	–	0.00025	210 ± 30	1.2 ± 0.05	0.00014	5
L414C	100 ± 3	–	–	0.01773	9.6 ± 0.5	2.5 ± 0.2	0.0008	5
L414D	20 ± 0.6	–	–	0.00618	1.1 ± 0.1	9.7 ± 1.7	0.001	5
L414K	370 ± 60*	120 ± 32 [45 ± 5]	590 ± 140 [55 ± 5]	0.0234	21 ± 2	3.5 ± 0.5	0.0022	4
L414V	330 ± 10	–	–	0.0009	41 ± 1	2.09 ± 0.07	0.00125	5
L414N	47 ± 4	–	–	0.0126	2.7 ± 0.4	12 ± 4	0.0009	6
L414A	41 ± 1	–	–	0.01228	3.8 ± 0.5	9 ± 3	0.00087	5
L414F	56 ± 2	–	–	0.0236	21 ± 2	3.5 ± 0.5	0.00112	5
L414Y	32 ± 2	–	–	0.02805	29 ± 2	2.7 ± 0.2	0.00076	4
L414I	920 ± 50	–	–	0.00408	145 ± 6	1.40 ± 0.04	0.00108	5
L414R	1600 ± 400	–	–	0.001	40000 ± 6000	0.30 ± 0.03	0.0007	3
N415A	38 ± 2	–	–	0.0082	13 ± 1	1.9 ± 0.2	0.0014	5
N415D	110 ± 5	–	–	0.00522	46 ± 1	1.6 ± 0.04	0.00098	5
N415E	2000 ± 70	–	–	0.0012	3.2 ± 0.07	3.6 ± 0.2	0.00096	4
N415F	1500 ± 50	–	–	0.0011	22 ± 2	2.1 ± 0.3	0.0008	4
N415G	3.6 ± 0.4	–	–	0.00984	5.9 ± 0.4	1.9 ± 0.2	0.00154	5
N415I	18000 ± 400*	3000 ± 800 [42 ± 5]	30000 ± 2000 [58 ± 5]	0.00605	290000 ± 80000	0.3 ± 0.6	0.0012	3
N415K	9900 ± 600	–	–	0.00938	35 ± 4	2.8 ± 0.2	0.00032	4
N415L	7800 ± 600	–	–	0.001	860 ± 90	0.99 ± 0.05	0.85608	2
N415Q	1900 ± 100	–	–	0.00736	29 ± 5	2.2 ± 0.2	0.00048	4
N415R	4700 ± 400	–	–	0.00594	18 ± 0.5	3.3 ± 0.1	0.00126	5
N415S	53 ± 3	–	–	0.00744	45 ± 6	1.4 ± 0.04	0.00112	5
N415W	3400 ± 70	–	–	0.00848	25 ± 0.6	1.1 ± 0.1	0.00064	4

*Indicates weighted time constant. Desensitization decays measured in pH 5. Recovery measured in pH 8.

To assess statistical differences in side chain angles from molecular dynamics simulations, we measured the median side chain angle from all three chains within each simulation as median is less sensitive to outliers (or multiple populations) than mean. To compare side chains angles between conditions (e.g., N415 angles for wild type vs. L414D), we compared the median side chain angles for each simulation in each group using a Mann–Whitney *U* test where n is the number of simulations.

## Results

### Kinetics of Desensitization and Recovery Are Strongly Influenced by Mutations to the β11-12 Linker

With the exception of the acidic pocket, the most salient difference between the resting and desensitized states of cASIC1 is the isomerization of the linker connecting the 11th and 12th beta strands. This is illustrated in Figure 1A where the side chains of the linker residues are shown within the palm domain as well as superimposed in the resting and desensitized states [PDB: 6VTL and 6VTK (Yoder and Gouaux, 2020)]. In the resting state, Leu414 points outward from the central axis of the channel and slightly up. However, in the desensitized state, the Leu414 side chain swings inward and down, toward a hydrophobic cleft (Rook et al., 2020). In parallel, Asn415 rotates from a down and inward position in the resting state to an upward and out position in the desensitized state. This swivel was proposed to act as a molecular clutch, uncoupling the proton sensing extracellular domain from the channel pore and thereby allowing the extracellular domain to adopt an “active-like” protonated conformation while the pore shuts closed (Yoder et al., 2018). Consistent with this, using non-canonical amino acid trapping, we recently demonstrated that local motion of this area is critical for desensitization to occur (Rook et al., 2020). We also found that slight mutations to the Leu414 position (i.e., L414A) result in dramatic changes in channel desensitization and recovery (Rook et al., 2020). The goal of the present study was to further probe the molecular determinants of this linker isomerization using molecular dynamics simulations.

A challenge in using molecular dynamics simulations to explore functional transitions of ion channels is the discrepant time scales of molecular dynamics simulations (∼10^–7^ s) vs. patch clamp experiments (∼10^–3^ s). In this case, the functional transitions of interest are desensitization and recovery, respectively. To partially bridge the timescale gap, we have previously taken advantage of the pH-dependence of recovery from desensitization (Rook et al., 2020). ASICs recover faster from desensitization when incubated in more alkaline solutions. This effect is quite strong in cASIC1, which recovers with a time constant of 840 ± 90 ms at pH 8 but accelerates to 7.5 ± 0.4 ms at pH 10 (Rook et al., 2020). By starting simulations from the desensitized/protonated crystal structure and de-protonating acidic residues (i.e., approximating a functional transition from extracellular pH 5 to 9), we can provoke the beginning of the transition from desensitized to resting state. When this strategy is combined with mutations which accelerate recovery from desensitization, we have previously been able to observe the linker flipping behavior directly in molecular dynamics simulations (Rook et al., 2020). Hence, we again focused on capturing the recovery process in the simulations, rather than the desensitization process.

A second challenge or caveat in comparing molecular dynamics simulations with results from patch clamp experiments is that the measured macroscopic channel desensitization and recovery processes reflect a population of channels transitioning between resting, active, pre-active and desensitized states. Changes to one or more of these transitions may alter the macroscopic desensitization decays measured in the population without much influence on the microscopic desensitization transition itself (Jones and Westbrook, 1996; Daniels et al., 2013). Since molecular dynamics simulations more likely reflects the single channel state transitions, we must bear this caveat in mind.

We set out to extend our previous work by first characterizing a wide range of mutations at both the Leu414 and Asn415 positions. The fastest mutations uncovered would then be used for molecular dynamics simulations to better probe the interactions governing linker isomerization. As an added benefit, these experiments would further map the structure-function relationships in this critical region.

To do this, we made four additional mutations to Leu414 in cASIC1 (D, C, K, and V). This isoform was used to enable direct comparison with past work, due to the larger number of structures and because, in our hands, cASIC1 undergoes far less tachyphylaxis [long-lived inactivation or run-down (Chen and Grunder, 2007)], thus enabling longer recordings of recovery from desensitization. To measure recovery, we excised patches from transfected ASIC KO HEK cells (Rook et al., 2021a) and used piezo-driven fast perfusion to jump from pH 8 to 5. Recovery was mapped using a standard two-pulse protocol where channels were first exposed to a conditioning pulse of pH 5 to populate the desensitized state followed by jumping to pH 8 for variable intervals before a test pulse of pH 5 is applied. Conditioning pulse durations were chosen for each mutation to achieve equilibrium desensitization. However, the test pulse was generally 500 ms in duration (although 50 ms was used for N415G). Strikingly, each mutation showed a statistically significant difference in recovery from desensitization compared to wild type (Figure 1C and Table 1). To our surprise, we found the L414D showed the fastest recovery from desensitization (τ = 1.1 ± 0.1 ms, *m* = 9.7 ± 1.7, *n* = 5) while the structurally smaller L414A and L414C were actually slower than L414D (Figures 1B,C). Even with this new expanded series, we could discern no clear pattern or structural feature that predicted fast recovery. The large and positively charged Arg side chain desensitized and recovered slowly (Rook et al., 2020), however, the smaller Lys side chain actually recovered faster than wild-type (τ = 21 ± 2 ms, *m* = 3.5 ± 0.5, *n* = 4). Moreover, the sizeable hydrophobic side chains of Tyr and Phe recovered faster.

**FIGURE 2:**
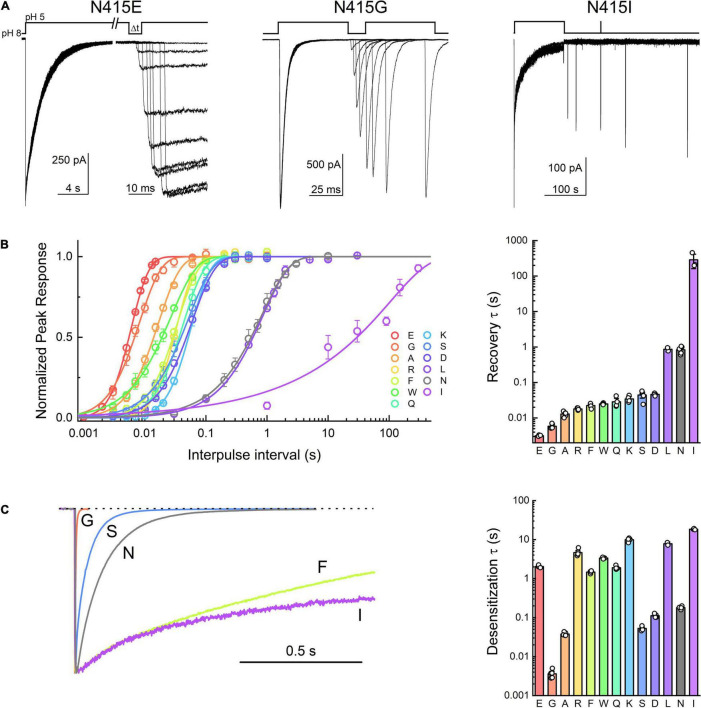
Acid-sensing ion channel (ASIC) desensitization and recovery is altered by mutations to Asn415. **(A)** Example outside-out patch recordings of N415E, G, and I (left, middle, and right, respectively). Note the change in time scale in the N415E example. **(B)** Summary recovery time course (left) and time constants (right) for Asn415 mutant panel. **(C)** Example traces (left) and summary of time constants (right) for desensitization decays of Asn415 mutant panel. Symbols represent individual patches and error bars are SEM. Differences in both desensitization and recovery kinetics for all mutations were statistically significant from wild type, with the exception of N415L recovery that was not different.

In the course of this experiment, we also measured the rates of entry into desensitization of the mutant panel during the conditioning pulse (Figure 1D). As with recovery, all mutations showed statistically significant differences in desensitization kinetics compared to wild type (Figure 1D and Table 1). We found that L414D was the fastest of the new mutations, with a desensitization time constant of 20 ± 0.6 ms (*n* = 5). Taken together, our data suggest that L414D is a promising mutation with extraordinarily fast kinetics, both desensitization and recovery, and hence may be a suitable candidate for molecular dynamics simulations. Specifically, using L414D to provoke swiveling might enable us to study the trajectory of the unaltered partner N415. To identify a similar candidate in N415 (fast mutation which would allow us to study the interactions of L414), we mutated N415 to 12 different residues and mapped their recovery from desensitization (Figures 2A,B). Among these, N415E showed the fastest recovery from desensitization (3.0 ± 0.7 ms, *m* = 3.6 ± 0.2, *n* = 4). While N415E recovered very quickly, this clone underwent desensitization slower than wild type (Figure 2) with a time constant of 2 ± 0.07 s. In contrast, N415G both entered and exited the desensitized state quickly (Figure 2) and hence was selected for subsequent molecular dynamics simulations. We found that nearly all mutations accelerated recovery in a statistically significant manner, while some slowed and others accelerated desensitization. The sole exception was N415I, which showed nearly 100 fold slower desensitization and recovery compared to wild type (Figure 2 and Table 1).

**FIGURE 3:**
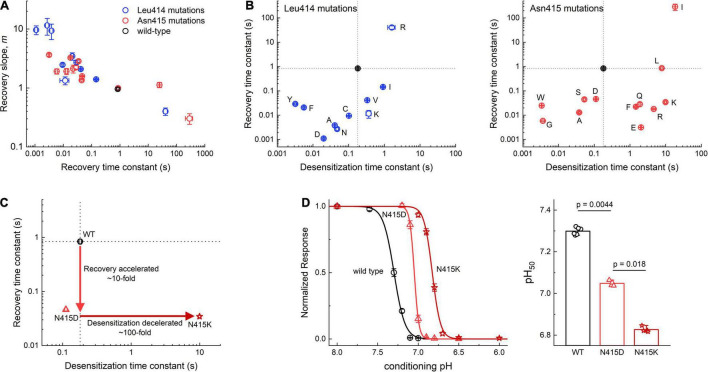
Summary of Leu414 and Asn415 mutant kinetics. **(A)** Recovery slope (*m*) vs. time constant (τ) of the recovery curve for mutant series in Leu414 (blue) or Asn415 (red). **(B)** Summary of entry and exit time constants for each mutant in L414 (left) and Asn415 (right). Note that the wild type side chains (black) are relative isolated with any deviation producing order of magnitude changes in kinetics. **(C)** N415D has ∼10 fold faster recovery with comparable desensitization vs. wild type. N415K has ∼100 fold slower desensitization with comparable recovery as N415D. **(D)** Steady-state desensitization curves for wild type, N415D and N415K (left) and pH_50_s from each patch. *P*-values are from randomization tests. All error bars are SEM.

Two additional general observations emerge from this mutant panel. First, we have previously remarked that when the time course of ASIC recovery is accelerated (by mutation or pH), the slope of the recovery curve becomes steeper and when recovery is slowed, the slope becomes more shallow (Rook et al., 2020). This positive correlation between rate constant of recovery and slope is also found within this expanded dataset (to our knowledge the largest panel of recovery experiments for ASICs) (Figure 3A). Such behavior is not expected for a simple kinetic model (Rook et al., 2020). A precise molecular explanation for this robust feature is unclear but its persistence through the mutant series is an essential aspect of ASIC recovery. Second, any alteration to Leu414 or Asn415 produces very large changes in kinetics. This is seen in the scatter plots in Figure 3B, where the time constants for desensitization are plotted in the abscissa and the recovery in the ordinate. It is immediately apparent that Leu414 and Asn415 are relatively isolated. That is, any small changes to these side chains produce robust, often order of magnitude, effects in desensitization and recovery. In principle, the kinetics of desensitization and recovery reflect the stability of the desensitized state. If all other transitions remain constant, a mutation causing slower desensitization (or faster recovery) should also lead to a less stable desensitized state, requiring stronger stimuli to desensitize. This should manifest as a right shift or acidic shift in the SSD curve, the process where ASICs desensitize upon exposure to acidic conditions too mild to detectably activate the channel. To examine this, we measured an SSD curve for N415D that has faster recovery than wild type with a comparatively small change in desensitization (Figure 3C). As expected, this mutation produced a less stable desensitized state as evidenced by the significant right shift in the SSD curve (wild type: pH_50_ 7.31 ± 0.01, n_*H*_ 6.6 ± 0.3, *n* = 6; N415D: pH_50_ 7.048 ± 0.005, n_*H*_ 15.9 ± 0.4, *n* = 4, *p* = 0.0044, Figure 3D). We next examined N415K, a mutation with comparable recovery to N415D but roughly 100 fold slower desensitization kinetics (Figure 3C). This mutation should produce a further right shift in the SSD curve. Indeed, as predicted the pH_50_ for SSD of N415K was significantly more acidic than N414D (N415K: pH_50_ 6.827 ± 0.005, n_*H*_ 8.7 ± 0.4, *n* = 4, *p* = 0.018 vs. N415D, Figure 3D). Our kinetic data (Figures 3A,B) underscores the essential nature of the β11-12 in governing transitions to the desensitized state. Furthermore, the remarkable sensitivity of this region suggests that the interactions surrounding this linker have been exquisitely shaped by evolution to obtain exactly the desired kinetic behavior (Figures 3A,B) or desensitized state stability (Figure 3D). To explore what these interactions might be, we turned to all-atom molecular dynamics simulations.

### Molecular Dynamics Simulations With Fast Recovering Mutations

**FIGURE 4:**
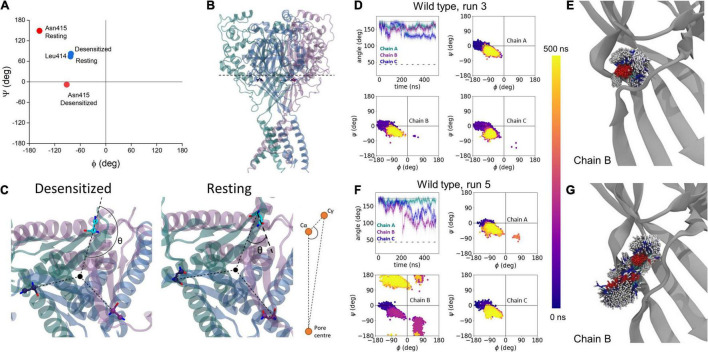
Analysis of transitions to resting state using molecular dynamics simulations. **(A)** Phi/psi plots of Leu414 (blue) and Asn415 (red) in desensitized and resting states. **(B)** Slice-through of the desensitized state structure to show the plane of angle defined in panel **(C)**. **(C)** Illustration of change in side chain angle, with respect to Cγ and channel center in desensitized and resting states. **(D)** Example of wild type run where no substantial changes were seen in the side chain angle (upper left), or phi/psi plots in each chain (upper right, lower panels). The dotted line illustrates the side chain angle of N415 in the desensitized state structure while the dashed line illustrates the side chain angle in the resting state structure. **(E)** Overlay of 1000 Asn415 positions at regular intervals over the 500 ns simulation. **(F,G)** Same as panels **(D,E)** but for wild type run where motion toward a resting-like conformation was observed in chain B.

To approximate the early phase of the desensitized to resting transition (i.e., the recovery process), we simulated either wild type, L414D or N415G desensitized state trimers in a bilayer. To accelerate conformational changes as illustrated previously (Rook et al., 2020), all acidic side chains were de-protonated in the simulations (approximating a pH of 8–9 based on pK_*a*_ estimates) and ensuing motions were examined. Each construct was simulated five times for 500 ns each. We used two main metrics to quantify changes observed during these simulations. First, we measured the phi and psi dihedral angles for residues 414 and 415 to quantify backbone conformational changes and flexibility (Figure 4A). However, since the phi/psi dihedral angles do not substantially change between resting and desensitized states for Leu414, we primarily used this metric for Asn415. Second, we measured the side chain angle, defined as the angle centered at the α carbon, between the channel center and the γ carbon atoms of Leu414 and Asn415 (Figures 4B,C). Examples of these metrics in wild type, one for a very stable simulation and one for a simulation showing some local instability, are shown in Figures 4D–G. In four out of five wild type simulations, there was minimal change in these metrics. Figures 4D,E shows an example of such a stable run where the side chain angles and the phi/psi plots for each chain remain close to that of the desensitized state (Figure 4D) and the side chain position does not move appreciably (Figure 4E). However, in run 5 Asn415 of chain B undergoes a significant conformational shift from a desensitized-like side chain angle and phi/psi plot to become closer to the resting state (Figure 4F). In this simulation, the Asn415 side chain is also far more mobile (Figure 4G). We therefore turned to analyzing our fast recovering mutants, expecting to see greater dynamics.

**FIGURE 5:**
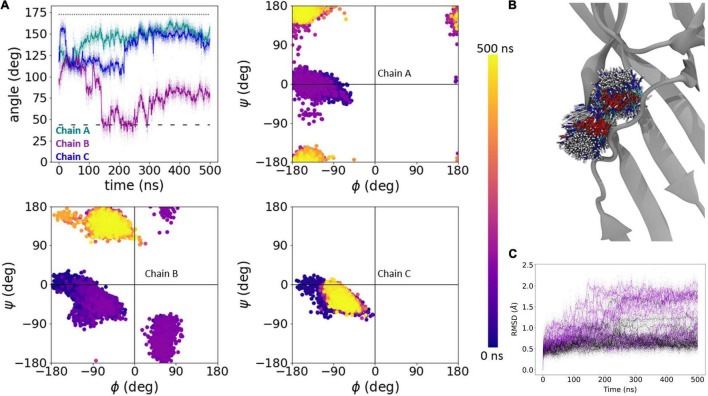
Dynamics of Asn415 in L414D mutation. **(A)** Side chain angle (upper left) and phi/psi plots (upper right, lower) of Asn415 chains in L414D simulation, run 2. The dotted line illustrates the side chain angle of N415 in the desensitized state structure while the dashed line illustrates the side chain angle in the resting state structure. **(B)** 1000 snapshots from chain B, at regular intervals, from simulation in panel **(A)**. **(C)** Calculated RMSD of individual chains as a function of time for Cα atoms of amino acid residues 409–420 of wildtype (black) and L414D mutant (violet). Raw data is plotted in the background as transparent dots, smoothed data is plotted as a solid line.

As expected of the fast recovering mutants, in all five L414D simulations we observed a much greater degree of Asn415 motion and a tendency for side chain angles and phi/psi plots to approach those of the resting state. Figure 5 shows one such example where at the start of the simulation, the Asn415 phi/psi plots of all three chains overlap with that expected of the desensitized structure (Figure 5A). Interestingly, the side chain angles begin to deviate quickly from that expected of the desensitized state. Around the 140 ns time point, a major rearrangement of chain B’s Asn415 side chain and backbone is evident both in the angle and phi/psi plots (Figure 5A). This change in conformation is further observed in a series of structural snapshots over the course of the simulation (Figure 5B). Comparing the root mean square deviation (RMSD) of the C_α_ -atoms of residues 409–420 for all simulated chains (three chains per protein times five repeats, i.e., 15 chains per system) moreover illustrates that while three to four chains in the wild type simulations undergo larger conformational changes (defined as reaching an RMSD > 1 Å), this is the case for 10 of the 15 chains for the L414D mutant (Figure 5C). Thus, looking at our full data set for wild type vs. the L414D mutant, it is also clear that the L414D point mutation provokes structural rearrangements to a larger degree than observed for the wild type.

**FIGURE 6:**
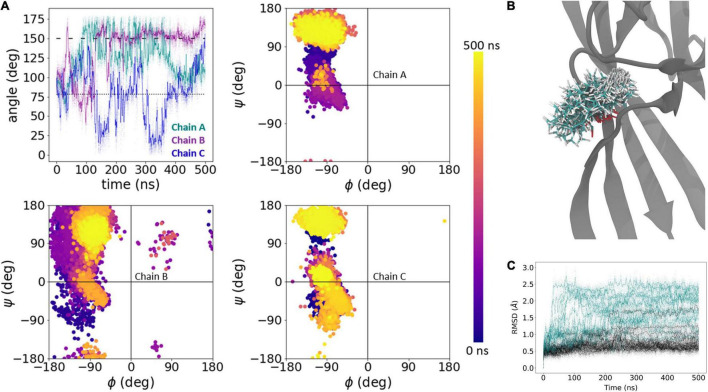
Dynamics of Leu414 in N415G mutation. **(A)** Side chain bond angle (upper left) and phi/psi plots (upper right, lower) of Leu414 chains in N415G simulation, run 2. The dotted line illustrates the side chain angle of L414 in the desensitized state structure while the dashed line illustrates the side chain angle in the resting state structure. **(B)** 1000 snapshots from chain B, at regular intervals, from simulation in panel **(A)**. **(C)** Calculated RMSD of individual chains as a function of time for Cα atoms of amino acid residues 409–420 of wildtype (black) and N415G (teal). Raw data is plotted in the background as transparent dots, smoothed data is plotted as a solid line.

We next conducted the same block of five 500 ns simulations of N415G (Figure 2) to examine the dynamics of Leu414 flipping. As with the L414D simulations, N415G resulted in an overall greater degree of motion of the Leu414 side chain as well as a highly increased backbone flexibility. This is illustrated with an example for a single chain in Figures 6A,B. As above, comparing the RMSD values for all chains for the β11-12 linker region, it is again clear that the mutation causes a much larger degree of structural change than observed for the wild type (14 out of 15 chains with an RMSD > 1 Å for the mutant vs. 3–4 chains for the wild type) (Figure 6C).

**FIGURE 7:**
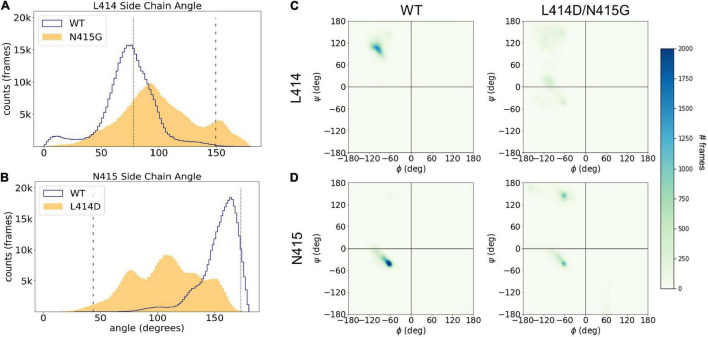
Leu414 and Asn415 are more dynamic in mutant compared to WT simulations. **(A)** Histogram of side chain angle of Leu414 in all WT (blue trace) and N415G simulations (yellow area). The dotted line illustrates the side chain angle of L414 in the desensitized state structure while the dashed line illustrates the side chain angle in the resting state structure. **(B)** Same measurement as in panel **(A)** but comparing Asn415 sidechain angle in WT (blue trace) and L414D (yellow area) simulations. **(C)** Frequency of phi/psi pairs for Leu414 in WT simulations (left) or N415G simulations (right). **(D)** Frequency of phi/psi pairs Asn415 in WT simulations (left) or L414D simulations (right).

In addition to analyzing the individual simulations, we also examined the overall distributions for the side chain angles as well as the phi/psi backbone angles for our full data set (Figure 7). The median side chain angles of L414 were statistically different between wild type and the “fast mutant” N415G (median L414 side chain angles of wild type ranged from: 67.1–79.3°; N415G: 81.4–120.3°, *n* = 5, *p* < 0.01, Mann–Whitney *U* test). In particular, the L414 side chain angle only shows one population for the wild type simulations. The population peak overlaps with the side chain angle in the crystal structure of the desensitized state (Figure 7A). Interestingly, in the N415G mutant the L414 side chain angle measurements clearly form two populations. One of these is at an angle slightly higher than the angle in the crystal structure for the desensitized state, while the other population almost matches the side chain angle of L414 in the crystal structure of the resting state. In the case of N415, a similar pattern appears. The median side chain angles of N415 were different between wild type and L414D simulations (median N415 side chain angles of wild type ranged from: 153.6–161.3°; L414D: 95.7–128.7°, *n* = 5, *p* < 0.01, Mann–Whitney *U* test). But whereas the wild type simulations predominantly show side chain angles tightly grouped close to that of the desensitization state structure (Figure 7B), in the L414D simulations multiple populations arise, all of which are closer to the resting state angle (Figure 7B). This data again highlight that the mutations appear to push the structure of the β11-12 linker closer to the resting state structure. When plotting the backbone dihedral angles for all our simulation data, it is obvious that the wild type simulations show one overall population for both L414 (Figure 7C) and N415 (Figure 7D), while for both of the mutants, the populations are less well defined, underlining the larger conformational flexibility caused by the point mutations.

**FIGURE 8:**
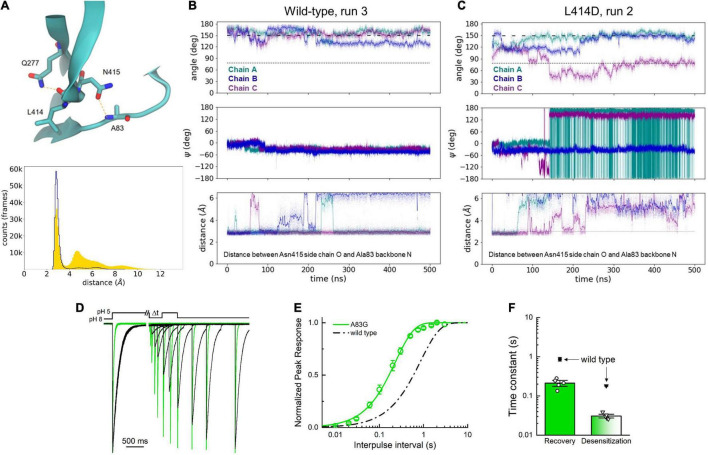
Asn415 and Ala83 interaction may influence desensitized state stability. **(A)** Depiction of Asn415-Ala83 interaction in the desensitized state (upper) and histogram of distances in wild-type (blue trace) and L414D (yellow area) simulations (lower). **(B)** Side chain angle (upper), Asn415 psi angle (middle) and distance between Asn415 side chain oxygen atom and Ala83 backbone nitrogen atom (lower) in a stable wild-type simulation. The dotted line illustrates the side chain angle of L414 in the desensitized state structure while the dashed line illustrates the side chain angle in the resting state structure. **(C)** Same as panel **(A)** but for an L414D simulation. **(D)** Example recovery from desensitization experiments for wild-type (black) and A83G (green). **(E)** Recovery from desensitization curves for wild-type (black) and A83G (green). **(F)** Summary of recovery (circles) and desensitization (triangles) time constants. Open symbols represent individual patches. Filled symbols represent mean data from Rook et al. (2020). Error bars are SEM.

We have previously described that the L414A mutation accelerates conformational changes and how L414 is nested in a hydrophobic pocket in the desensitized state (Rook et al., 2020). Thus, it is perhaps not surprising that the L414D mutation also increases the flexibility of the β11-12 linker. On the contrary, it is not immediately clear why the N415G mutant illustrates dramatically increased flexibility of the linker region. It is possible that these observed dynamics for the N415G mutant result from either the increased backbone flexibility expected with a Gly replacement or from the loss of some specific contacts of the Asn415 side chain when changing this position to N415G. To gain some insight into this question, and further analyze our wild type and L414D simulations, we examined this data set for candidate interactions, which may play roles in linker flipping. We observed that the backbone oxygen atom of Leu414 is frequently stabilized by a presumptive hydrogen bond interaction with the side chain of Gln277. This interaction has been reported in our prior simulations and plays a functional role in stabilizing the desensitized state (Rook et al., 2021a). We also observed a putative hydrogen bond between the side chain oxygen atom of Asn415 and the backbone amide N-H of Ala83 (Figure 8A). If this Asn415-Ala83 interaction has functional importance, one expects the interaction to be disrupted in simulations with faster recovering mutations. To test this, we measured the distance between the side chain oxygen atom of Asn415 and the backbone amide of Ala83 for all wild type and L414D simulations. As seen in Figure 8A (lower panel), simulations with the faster recovering mutation showed a substantial increase in the distance between these two atoms, well beyond the 3–3.5 Å of typical hydrogen bond interactions. We further examined the temporal dynamics of this interaction. As seen in Figure 8B with a stable wild type simulation, when the side chain angle and phi/psi plots remain at the desensitized state values, there is minimal change in the Asn415-Ala83 distance. However, when a fast recovering L414D trajectory is examined (Figure 8C), the Asn415-Ala83 distance increase is temporally correlated with the side chain angle and phi/psi dihedral angle changes. Thus, we hypothesized that the Asn415-Ala83 interaction is functionally important for stabilizing the desensitized state.

To test this experimentally, we substituted Ala83 for Gly. Standard mutagenesis cannot remove the Ala83 backbone amide, however, we reasoned that replacing the Ala with a Gly would impart additional flexibility, enabling the A83G position to move out of hydrogen bond distance from the Asn415, thereby weakening the interaction and accelerating recovery from desensitization. Consistent with this hypothesis, A83G resulted in marked acceleration of recovery (recovery τ of 210 ± 25 ms, *n* = 5, *p* < 0.001 vs. wild type, Figure 8). Taken together, these data support that the β11-12 linker flipping is critical for ASIC desensitization and highlight a new interaction between the Ala83 backbone and the side chain of Asn415. Our work also advances several metrics for analyzing ASIC simulations in the future.

## Discussion

### Comparison With Past Work

There is considerable evidence indicating that the β11-12 linker is the essential structural determinant of pH-induced desensitization. Prior to the availability of ASIC structures, functional studies reported strong effects of mutations to the area surrounding the β11-12 linker (Coric et al., 2003). As structures of different states emerged, complimentary functional work supported the critical nature of the β11-12 linker in channel desensitization (Li et al., 2010a,b; Roy et al., 2013; Wu et al., 2019). Work from our own labs using UV-reactive non-canonical amino acid trapping has shown that desensitization can be nearly abolished by preventing the isomerization of the β11-12 linker (Rook et al., 2020), supporting the idea that linker swivel is necessary for desensitization. Here we examine a large panel of mutations to this linker and find that virtually every single mutation produces at least an order of magnitude shift in desensitization or recovery time constants (Figures 1–3). The marked sensitivity of this area to subtle perturbations is further evidence supporting the critical nature of this linker and the general “molecular clutch” model. However, is the β11-12 linker isomerization the only mechanism for channel desensitization?

Mutations in the thumb domain influence desensitization kinetics (Krauson et al., 2013; Krauson and Carattino, 2016; Braun et al., 2021) as does simple extracellular anion substitution (Kusama et al., 2010, 2013). How can these data be reconciled with a model where linker flipping is the crucial feature governing ASIC desensitization? Structural studies reveal an anion binding site physically proximal to the linker (15 Å from the Cl^–^ anion to the Cα of Leu414 in PDB 2QTS) (Jasti et al., 2007). This anion site is formed by Arg310 and Glu314 in the thumb domain and Lys212 from the adjacent subunit (cASIC1 numbering). Lys212 resides in a peptide loop immediately above the β11-12 linker. It is plausible that anion occupancy exerts an allosteric influence on linker flipping by stabilizing or destabilizing this upper loop through interactions between the anion and Lys212. Further, given that the base of the thumb forms the anion binding site, mutations to the thumb may impact desensitization kinetics by virtue of influencing anion occupancy. Clearly, further work is needed to test this hypothesis, however, it is certainly possible that the β11-12 linker represents a final common pathway for channel desensitization.

### Physiological Role of Desensitization

**FIGURE 9:**
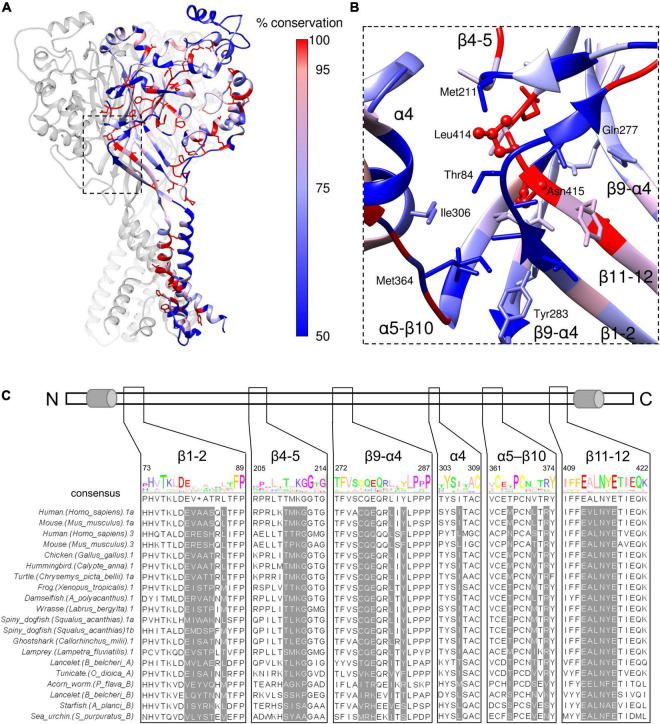
β11-12 linker is highly conserved while the surrounding region is not. **(A)** Resting state structure (PDB: 6VTL) with one subunit colored based on sequence conservation using sequence alignment from ASIC sequences in panel **(C)**. Side chains of completely conserved positions are shown. **(B)** Close up view of the boxed region in panel **(A)** using the same coloring. Any amino acids within 5 Å of Leu414 or Asn415 have side chains depicted. **(C,** upper) Schematic of ASIC1 linear peptide with cylinders indicating transmembrane domains 1 and 2. (**C**, lower) Multi sequence alignment of the indicated ASICs. The amino acid regions shown from the alignment correspond to the segments in panel **(B)**. Positions highlighted in gray are within 5 Å of Leu414 or Asn415 and have side chains depicted in panel **(B)**.

The β11-12 linker is highly sensitive to mutation, as is the surrounding area (Coric et al., 2003; Wu et al., 2019; Rook et al., 2020, 2021b) with even conservative mutations producing log unit shifts in kinetics. This region is also highly conserved within ASICs. We aligned amino acid sequences of acid-responsive ASIC variants from mouse and human (ASIC1a and 3) as well as ASIC1a-like sequences from chicken, hummingbird, two amphibians, several fish, spiny dogfish (Springauf and Grunder, 2010) along with more distant sequences known to be pH-responsive (Lynagh et al., 2018). The percentwise conservation determined from this alignment was used to color the cASIC1 resting state subunit (Figure 9A). As seen in the expanded view of the structure (Figure 9B), and the alignment itself (Figure 9C), the Leu414 and Asn415 residues were completely conserved and the remaining positions in the β11-12 linker were highly conserved. The conservation of these critical positions suggest that the core mechanism of desensitization, or some related function, is physiologically important and has been preserved for hundreds of millions of years. However, the kinetics of ASIC desensitization vary considerably depending on the species and subunit variants under investigation. If the β11-12 linker is nearly completely conserved, what might be the source of species and subunit specific kinetics? Interestingly, the regions surrounding the β11-12 linker are not as well conserved. From the point of view in Figure 9B, the β11-12 linker is surrounded by the β1-2 and β4-5 linkers on the “front” and “top” faces, respectively, as well as the β9-α4 linker on the “right” side. Residues from the β9-α4 and α5-β10 linkers of the adjacent subunits surround the Leu414 and Asn415 on the “bottom” and “left” faces, respectively. Likewise, the α4 helix of the thumb domain of the adjacent subunit is near the β11-12 linker (Figure 9B). To further highlight the potential influence of these less conserved regions, we determined which side chains are within 5 Å of Leu414 and Asn415 in either resting or desensitized states (PDB:6VTL and 6VTK) and show these side chains in Figure 9B. These positions are also highlighted in the alignment in Figure 9C. Several of these highlighted positions have previously been shown to alter ASIC kinetics (Coric et al., 2003; Cushman et al., 2007; Li et al., 2010b; Roy et al., 2013). Therefore, we suggest that the main source of subunit or species specific kinetics is not the β11-12 linker itself but rather the surrounding amino acid residues which influence the β11-12 linker isomerization. Mutations to the surrounding area may lead to smaller changes in kinetics, more suitable for tuning receptor gating compared to the rather large shifts produced by mutating the β11-12 linker directly.

Our functional work focuses on the kinetics of desensitization and recovery, however, it is possible that the more physiologically important outcome is SSD. Acidic stimuli which are not strong enough to detectably activate the channel can still desensitize it (i.e., pH 7.1) (Grunder and Pusch, 2015). Thus, SSD is observed right near physiological pH values of 7.4, depending on the subunit and species. Moreover, the pH-dependence of SSD is modulated by numerous physiologically relevant signals such as extracellular Ca^2+^, FMR amide-related peptides, Cl^–^ as well as by the heteromeric composition of the channel itself (Babini et al., 2002; Sherwood and Askwith, 2008; Kusama et al., 2013). The SSD process provides a powerful mechanism to control the magnitude of ASIC currents at any one time. A slightly alkaline or acidic extracellular solution will increase or decrease the maximum possible ASIC response. Furthermore, this effect can then be influenced by local Ca^2+^, FMRF or Cl^–^ concentrations. Mutations that change desensitization or recovery kinetics will also alter the stability of the desensitized state, which in turn must impact the pH mid-point of the SSD curve (Rook et al., 2021a). This is seen in Figure 3 where mutations which accelerate recovery (N415D) or both accelerate recovery and slow desensitization (N415K) produce increasingly more acidic pH_50_ values for SSD, consistent with a destabilized desensitized state. Thus, it is possible that the conservation of the β11-12 linker, and alterations of the surrounding areas, arose because of the physiological role of SSD rather than the precise timing of ASIC desensitization and recovery kinetics. It is also possible that the kinetics of channel desensitization and recovery are important, however, mainly in the context of slow changes in extracellular pH as have been recently examined (Alijevic et al., 2020) or intracellular conformational changes which might be involved in signaling cascades (Wang et al., 2015, 2020; Couch et al., 2021). Understanding these issues will require a clearer accounting of the spatial-temporal dynamics of pH change *in situ* as well as introduction of ASICs with biophysically impactful mutations to appreciate the consequences.

## Data Availability Statement

The raw data supporting the conclusions of this article will be made available by the authors, without undue reservation.

## Author Contributions

MR, AA, MM, and DM designed and performed the experiments and simulations, analyzed the data, and interpreted the results. MM and DM obtained the funding. DM conceived the study and wrote the manuscript with input from all authors. All authors approved the final version of the manuscript.

## Conflict of Interest

The authors declare that the research was conducted in the absence of any commercial or financial relationships that could be construed as a potential conflict of interest.

## Publisher’s Note

All claims expressed in this article are solely those of the authors and do not necessarily represent those of their affiliated organizations, or those of the publisher, the editors and the reviewers. Any product that may be evaluated in this article, or claim that may be made by its manufacturer, is not guaranteed or endorsed by the publisher.
